# Influencing the properties of dysprosium single-molecule magnets with phosphorus donor ligands

**DOI:** 10.1038/ncomms8492

**Published:** 2015-07-01

**Authors:** Thomas Pugh, Floriana Tuna, Liviu Ungur, David Collison, Eric J.L. McInnes, Liviu F. Chibotaru, Richard A. Layfield

**Affiliations:** 1 School of Chemistry, The University of Manchester, Oxford Road, Manchester M13 9PL, UK; 2 Photon Science Institute, The University of Manchester, Oxford Road, Manchester M13 9PL, UK; 3 Division of Quantum and Physical Chemistry, Katholieke Universiteit Leuven, Celestijenlaan 200F, Leuven 3001, Belgium

## Abstract

Single-molecule magnets are a type of coordination compound that can retain magnetic information at low temperatures. Single-molecule magnets based on lanthanides have accounted for many important advances, including systems with very large energy barriers to reversal of the magnetization, and a di-terbium complex that displays magnetic hysteresis up to 14 K and shows strong coercivity. Ligand design is crucial for the development of new single-molecule magnets: organometallic chemistry presents possibilities for using unconventional ligands, particularly those with soft donor groups. Here we report dysprosium single-molecule magnets with neutral and anionic phosphorus donor ligands, and show that their properties change dramatically when varying the ligand from phosphine to phosphide to phosphinidene. A phosphide-ligated, trimetallic dysprosium single-molecule magnet relaxes via the second-excited Kramers' doublet, and, when doped into a diamagnetic matrix at the single-ion level, produces a large energy barrier of 256 cm^−1^ and magnetic hysteresis up to 4.4 K.

## Abstract (web summary)

Ligand design contributes to dictating the magnetic properties of lanthanide-based single-molecule magnets. Here, the authors report a series of phosphorus-ligated dysprosium complexes, and show that the dynamic magnetic properties change as the ligand is varied from phosphine to phosphide to phosphinidene.

## Graphical abstract

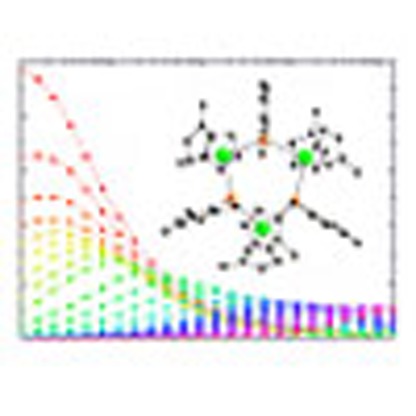

The observation of slowly relaxing magnetization in lanthanide bis(phthalocyanine) complexes, LnPc_2_, has made tremendous impact in molecular magnetism^1^,^2^. Subsequently, hundreds of lanthanide single-molecule magnets (Ln-SMMs) have been reported^3^,^4^,^5^, with many examples showing very large effective energy barriers to reversal of the magnetization (*U*_eff_)^6^,^7^. Some Ln-SMMs have also been developed for applications in nanoscale devices by, for example, deposition of TbPc_2_ onto carbon nanostructures or metallic surfaces^8^,^9^,^10^,^11^,^12^. The LnPc_2_ SMMs demonstrated that very large *U*_eff_ values can occur in monometallic complexes, and this important observation inspired the development of other monometallic Ln-SMMs with ligands such as polyoxometallates^13^ and organometallic ligands such as cyclo-octatetraene^14^,^15^,^16^. Organometallic synthesis offers an alternative strategy for designing Ln-SMMs owing to the diverse range of hard and soft ligands that can be accessed, and such ligands introduce new possibilities for influencing and enhancing the SMM properties^17^,^18^.

Phosphorus donor ligands provide opportunities for systematically modifying the properties of SMMs because their chemistry can be developed with neutral phosphines (R_3_P), mono-anionic phosphide (R_2_P^−^) and di-anionic phosphinidene (RP^2−^) ligands, as well as the tri-anionic phosphide ligand (P^3−^)^19^. Since electrostatic interactions strongly influence the electronic structure of lanthanides, the ability to synthesize compounds with similar molecular structures, but where the ligands carry different formal charges, could allow new ways of designing SMMs. Changing the organo-phosphorus ligand should also influence the exchange interactions in polymetallic systems, which is important because exchange is known to influence relaxation phenomena^3^,^4^,^5^. Phosphinidene complexes of the lanthanides are rare^20^,^21^,^22^,^23^,^24^,^25^, and, although their reactivity has been studied, their influence on 4f electronic structure has not. Indeed, phosphorus-ligated Ln-SMMs are unknown, hence we now target the SMM properties of dysprosium complexes with bridging phosphide and phosphinidene ligands. Here we show that organo-phosphorus chemistry can be used to influence the dynamic magnetic properties of lanthanide complexes; our observations have general implications for how main group organometallic chemistry can be used to develop new SMMs.

## Results

### Synthesis and structural studies

**Figure 1:**
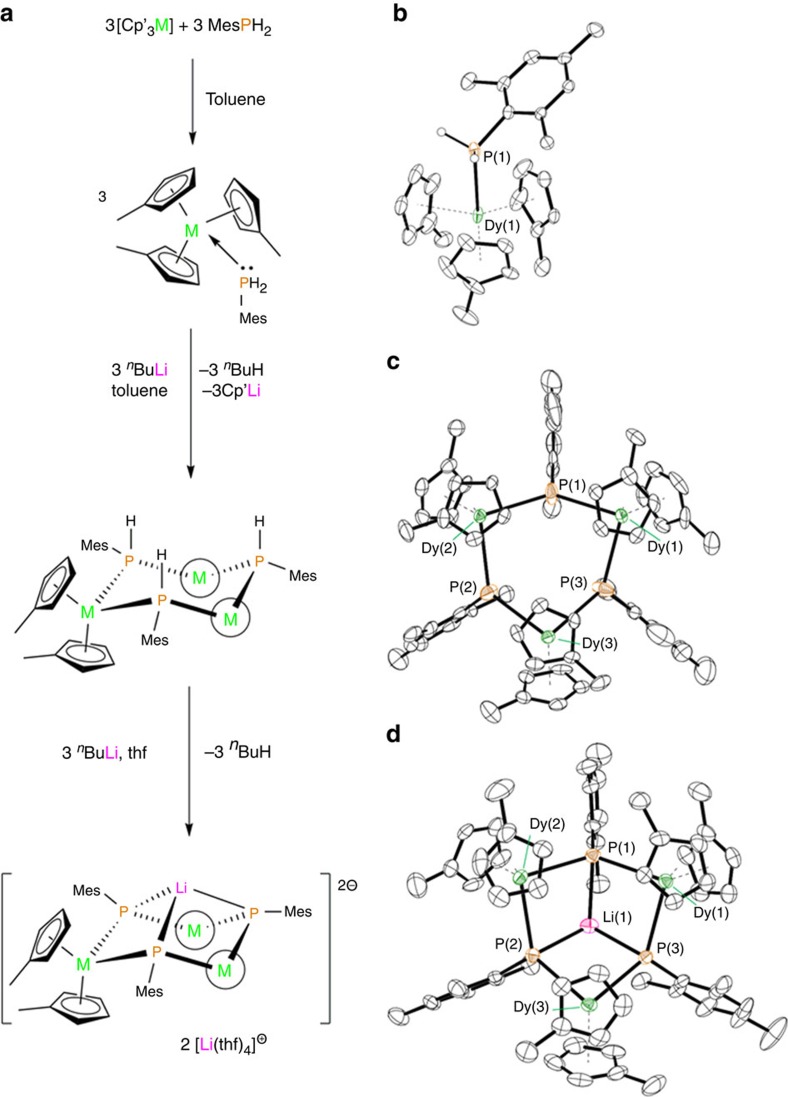
Synthesis and molecular structures. (**a**) Synthesis of the yttrium-phosphorus and dysprosium-phosphorus complexes. (**b**) Thermal ellipsoid representation of the molecular structure of **1-Dy**. (**c**) Molecular structure of **2-Dy**. (**d**) Molecular structure of **3-Dy**. Hydrogen atoms are omitted for clarity.

We initially targeted a primary phosphine complex of dysprosium and aimed to sequentially deprotonate the P–H bonds. The adduct [Cp′_3_Dy←PH_2_Mes] (**1-Dy**) (Cp′=η^5^-C_5_H_4_Me, Mes=mesityl) was synthesized by adding mesitylphosphine to tris(methylcyclopentadienyl)dysprosium (Fig. 1). Deprotonation of **1-Dy** by ^*n*^BuLi gave the trimetallic phosphide-bridged complex [(Cp′_2_Dy){*μ*-P(H)Mes}]_3_·toluene (**2-Dy**·toluene). Deprotonation of the P–H bonds in **2-Dy** by ^*n*^BuLi in thf produced [Li(thf)_4_]_2_[(Cp′_2_Dy)_3_(*μ*-PMes)_3_Li]·thf, which contains the phosphinidene-bridged trimetallic complex [(Cp′_2_Dy)_3_(*μ*-PMes)_3_Li]^2−^ (**3-Dy**). The analogous yttrium complexes [Cp′_3_Y←PH_2_Mes] (**1-Y**), [(Cp′_2_Y){*μ*-P(H)Mes}]_3_·toluene (**2-Y**·toluene) and [Li(thf)_4_]_2_[(Cp′_2_Y)_3_(*μ*-PMes)_3_Li]·thf, [Li(thf)_4_]_2_[**3-Y**]·thf, were synthesized in an identical manner. The molecular structures of all compounds were determined by X-ray diffraction; the main features of **1-Dy**, **2-Dy** and **3-Dy** (Fig. 1) are described below, with further details provided in Supplementary Tables 1–3, Supplementary Figs 1–10 and Supplementary Data 1–6.

Compound **1-Dy** (Fig. 1) contains a dysprosium centre coordinated to three η^5^-Cp′ ligands and a MesPH_2_ ligand, hence the dysprosium coordination geometry can be regarded as pseudo-tetrahedral with respect to the centroid positions of the Cp′ ligands. The Dy–P bond length is 3.009(1) Å and the Dy–C bond lengths are 2.676(7)–2.744(6) Å (average 2.710 Å). To the best of our knowledge **1-Dy** and **1-Y** are the first crystallographically characterized rare-earth complexes of primary phosphine ligands.

Compound **2-Dy** (Fig. 1) is a cyclic trimer in which the three {Cp′_2_Dy} units are bridged by μ-(mesityl)phosphide ligands, resulting in Dy–P bond lengths of 2.926(6)–2.951(6) Å. The Dy–Cp′ bond lengths in **2-Dy** fall in a broad range of 2.60(2)–2.72(1) Å (average 2.64 Å). The {Dy_3_P_3_} core of **2-Dy** adopts a chair-like conformation, in which each dysprosium is coordinated by two Cp′ ligands, with the mesityl substituents being oriented almost perpendicular to the P_3_ plane. It was not possible to locate the hydrogen atoms in the structure of **2-Dy**, but the P–H stretching vibrations occur in the IR spectrum at 2,358 and 2,318 cm^−1^ (Supplementary Fig. 8). The structure of **3-Dy** also consists of a chair-like Dy_3_P_3_ core but with a central lithium cation, in which the μ_3_-phosphinidene ligands bridge between dysprosium and lithium. The Dy–P bonds in **3-Dy** are 2.7850(15)–2.8249(15) Å, making them 0.13 Å shorter, on average, compared with the analogous distances in **2-Dy**, as expected based on stronger electrostatic bonding. The Dy–C bond lengths in **3-Dy** are longer, on average, by 0.05 Å compared with those in **2-Dy**, suggesting that the Cp′ ligands in **3-Dy** exhibit a degree of flexibility to accommodate the more compact {Dy_3_(PMes)_3_Li} core. The lithium cation occupies a position 0.896(8) Å out of the P_3_ plane, with Li–P bond lengths of 2.472(9), 2.477(10) and 2.557(8) Å, respectively. A mesityl *ortho*-methyl group may interact with lithium in **3-Dy** via an agostic bond; the Li(1)···C(45) distance of 2.813(11) Å is markedly shorter than the Li(1)···C(52) and Li(1)···C(63) distances of 3.318(10) and 3.452(10) Å, respectively. A structural feature that **3-Dy** shares with other rare-earth phosphinidene complexes is the bridging coordination mode of the ligand^20^,^21^,^22^,^23^,^24^,^25^.

### Magnetic property measurements

The static-field (d.c.) magnetic properties of polycrystalline **1-Dy**, **2-Dy**·toluene and [Li(thf)_4_]_2_[**3-Dy**]·thf were measured on a superconducting quantum interference device magnetometer in the temperature range of 1.8–300 K. At 300 K, the value of *χ*_M_ *T*(*T*) for **1-Dy**, where *χ*_M_ is the molar magnetic susceptibility, is 12.85 cm^3^ K mol^–1^; cooling the sample to 1.8 K produces a gradual decrease in *χ*_M_*T*, such that a value of 7.93 cm^3^ K mol^–1^ is eventually reached (Supplementary Fig. 11). The field (*H*) dependence of the magnetization (*M*) for **1-Dy** reveals a steep increase up to 1 T, followed by a more gradual increase up to 7 T, where the value of *M*=5.09 *μ*_B_ is consistent with a single Dy^3+^ ion with a ^6^H_15/2_ ground state (Supplementary Fig. 12). The *χ*_M_*T* values for **2-Dy**·toluene and [Li(thf)_4_]_2_[**3-Dy**]·thf at 300 K are 40.08 and 42.41 cm^3^ K mol^–1^, respectively, both of which are close to the predicted values for three uncoupled Dy^3+^ centres^26^; both *χ*_M_*T* values decrease gradually down to about 50 K, and then more rapidly to reach 17.81 and 19.47 cm^3^ K mol^–1^, respectively, at 1.8 K (Supplementary Fig. 11). For **2-Dy**·toluene and [Li(thf)_4_]_2_[**3-Dy**]·thf, the magnetization at 1.8 K increases rapidly up to about 2 T, before following a more gradual increase up to 7 T, where values of *M*=16.10 and 16.13 *μ*_B_ are reached. The *M*(*H*) data for the two trimetallic complexes are consistent with the expected value for three uncoupled Dy^3+^ ions (Supplementary Fig. 12).

**Figure 2:**
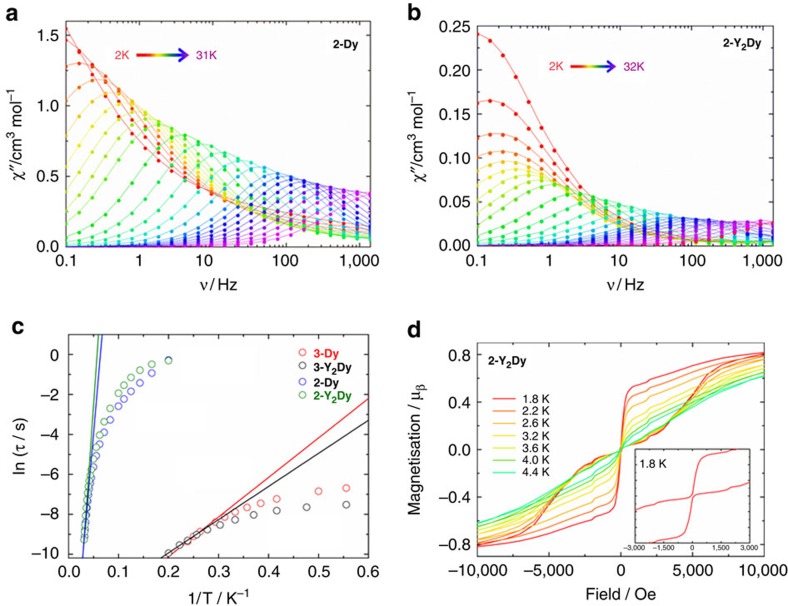
Magnetic properties of selected dysprosium-phosphorus compounds. (**a**) *χ*′′(*ν*) for **2-Dy**·toluene in the temperature range of 2–31 K with an a.c. field of 1.55 Oe and zero d.c. field. (**b**) *χ*′′(*ν*) for **2-Y**_**2**_**Dy**·toluene in the temperature range of 2–32 K with an a.c. field of 1.55 Oe and zero d.c. field. (**c**) Arrhenius plots for **2-Dy**·toluene (blue circles), **2-Y**_**2**_**Dy**·toluene (green circles), [Li(thf)_4_]_2_[**3-Dy**]·thf (red circles) and [Li(thf)_4_]_2_[**3-Y**_**2**_**Dy**]·toluene (black circles). The solid lines represent data fits in the range of 27–31 K for **2-Dy**·toluene, 28–32 K for **2-Y**_**2**_**Dy**·toluene, 3.8–4.2 K for [Li(thf)_4_]_2_[**3-Dy**]·thf and 3.9–5 K for [Li(thf)_4_]_2_[**3-Y**_**2**_**Dy**]·thf. (**d**) *M*(*H*) loops for **2-Y**_**2**_**Dy** 1.8–4.4 K with a sweep rate of 2.6 mT s^–1^.

The dynamic (a.c.) magnetic susceptibility properties of the three dysprosium compounds show much greater variation. For **1-Dy**, a maximum was observed in the plot of the out-of-phase magnetic susceptibility (*χ*′′) versus a.c. frequency (*ν*) at 1.8 K, but not at higher temperatures up to the maximum frequency that can be achieved with our susceptometer (Supplementary Fig. 13). These data indicate rapid relaxation of the magnetization in **1-Dy** above 1.8 K. In contrast, in zero applied d.c. field, the *χ*′′(*ν*) isotherms of **2-Dy**·toluene have well-defined frequency-dependent maxima up to 31 K, which demonstrates SMM behaviour with a large thermal energy barrier (Fig. 2 and Supplementary Figs 14 and 15). The energy barrier for the thermal relaxation in **2-Dy**·toluene was extracted using the relationship *τ*=*τ*_0_ exp (*U*_eff_/*k*_B_*T*), which gave *U*_eff_=210(6) cm^−1^, with a pre-exponential factor of *τ*_0_=6.53 × 10^–9^ s. Below about 20 K, the dependence of ln *τ* on 1/*T* weakens and deviates from linearity, but does not become fully temperature independent even at 1.8 K (Fig. 2). This observation implies that other relaxation mechanisms, such as quantum tunnelling of the magnetization and Raman or direct relaxation, occur concurrently with the thermal relaxation process at lower temperatures. In contrast, the *χ*′′(*ν*) isotherms in [Li(thf)_4_]_2_[**3-Dy**]·thf only show maxima below 3.6 K, and the position of the maximum moves only slightly as the temperature is reduced to 1.8 K (Supplementary Figs 16 and 17). The plot of ln *τ* versus 1/*T* for the phosphinidene-bridged complex produces an anisotropy barrier of *U*_eff_=13(1) cm^−1^ (*τ*_0_=7.75 × 10^–7^ s), that is, more than an order of magnitude smaller than in phosphide-bridged **2-Dy**·toluene.

To examine the influence of dysprosium–dysprosium interactions, magnetically dilute samples of 5% dysprosium in **2-Y**·toluene and [Li(thf)_4_]_2_[**3-Y**]·thf were prepared; dilution was achieved by mixing **1-Y** and **1-Dy** in a 20:1 ratio and then synthesizing the complexes according to Fig. 1. This enabled isolation of crystalline samples of **2-Y**_**2**_**Dy**·toluene in a matrix of **2-Y**·toluene and, similarly, [Li(thf)_4_]_2_[**3-Y**_**2**_**Dy**]·thf in a matrix of [Li(thf)_4_]_2_[**3-Y**]·thf. For both doped trimetallic compounds the a.c. susceptibility data are similar to those measured for the pure samples (Fig. 2, Supplementary Figs 18 and 19), that is, the phosphinidene-bridged complex shows very weak SMM behaviour, while **2-Y**_**2**_**Dy**·toluene is an SMM but with an increased anisotropy barrier of *U*_eff_=256(6) cm^−1^. There is a much clearer and more important difference in the *M*(*H*) hysteresis measurements for the undiluted and diluted systems. For **2-Dy**·toluene, the *M*(*H*) curve at 1.8 K (Supplementary Fig. 20) shows very narrow hysteresis, while hysteresis is found for **2-Y**_**2**_**Dy**·toluene up to 4.4 K, with significant widening of the butterfly-shaped loops as the temperature is lowered (Fig. 2). The coercive field for **2-Y**_**2**_**Dy**·toluene at 1.8 K is estimated to be *H*_c_≈300 Oe, with a small remanent magnetization of *M*_r_≈0.03 *μ*_B_ (sweep rate 2.6 mT s^–1^). The *M*(*H*) plot for **2-Y**_**2**_**Dy**·toluene also shows steps at *H*≈±1,800 Oe, which is probably due to small amounts of the partially doped di-dysprosium complex **2-YDy**_**2**_. In low magnetic fields, the ground state of **2-YDy**_**2**_ is defined by antiferromagnetic exchange, but above a certain field strength the ground state changes to ferromagnetic and has a greater magnetic moment, hence the steps in the *M*(*H*) curves. The precise field at which the steps are found is consistent with the calculated exchange spectrum from **2-Dy** (see below). A feature similar to this has been observed in magnetic dilution studies of di-dysprosium SMMs^27^,^28^. As *U*_eff_ is similar for **2-Dy**·toluene and **2-Y**_**2**_**Dy**·toluene, the difference in hysteresis must be due to non-thermal relaxation being more important in the undiluted compound.

### Quantum chemical calculations

Further insight into the electronic structure and the magnetic blocking in the dysprosium complexes was obtained using *ab initio* quantum chemical calculations. Current *ab initio* methods are not suitable for treating several magnetic 4f centres simultaneously; therefore, in the case of **2-Dy** and **3-Dy** appropriate fragmentation was imposed, hence Lu^3+^ was used in place of neighbouring Dy^3+^ (Supplementary Figs 22–24). All calculations on mono-dysprosium fragments were performed with MOLCAS^29^ and were of CASSCF/RASSI/SINGLE_ANISO type (Supplementary Tables 4–9)^30^. Tables 1 and 2 show the energy spectrum and the magnetic anisotropy of the lowest Kramers' doublets on individual dysprosium sites of the three compounds.

For the phosphine adduct **1-Dy**, the lack of SMM properties is consistent with the absence of strong magnetic axiality of the ground and excited doublets (Table 2). The presence of large *g*_x,y_ values in the ground state enables fast quantum tunnelling between states with opposite magnetization, destroying any potential blocking of the magnetization^31^,^32^. This can be rationalized in terms of the molecular and electronic structure of **1-Dy**, where the main magnetic axis (*g*_z_) lies in a plane defined by three Cp′ ligands (Supplementary Fig. 26). The electrostatic potential of the ligand field is dominated by the anionic Cp′ ligands, which are much closer to dysprosium (average Dy–C=2.710 Å) than the charge-neutral phosphine ligand (Dy—P=3.009 Å). Strong equatorial ligand fields are known to diminish magnetization blocking in Dy^3+^ complexes^14^,^33^, hence the weak SMM properties of **1-Dy**.

**Figure 3:**
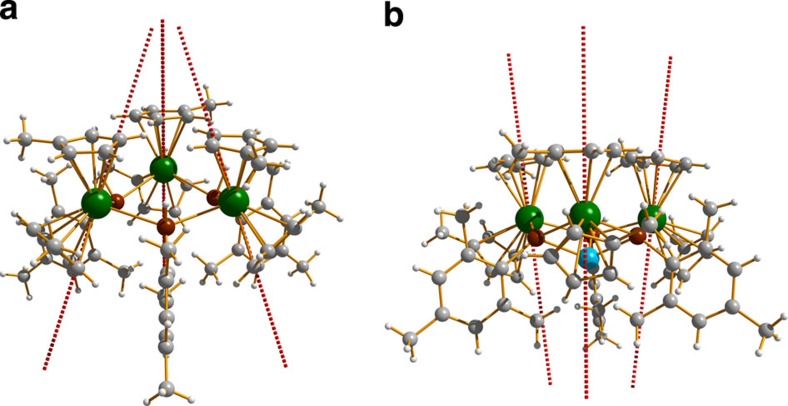
Orientation of the main magnetic axes in the ground Kramers' doublets. (**a**) For **2-Dy**. (**b**) For **3-Dy**. The magnetic axes are represented as dashed red lines. The dysprosium centres are represented as green spheres and the phosphorus atoms as orange spheres.

The dysprosium sites in **2-Dy** display the strongest magnetic axiality in the ground and excited doublet states, which is combined with the largest splitting of the eight Kramers' doublets within the ground *J*=15/2 manifold of the three systems investigated. The local main magnetic axes (*g*_z_) in **2-Dy** make angles of 69°–72° with the Dy_3_ plane (Fig. 3, Table 2). This direction of the magnetization axis on each dysprosium is the result of the strong axial ligand field generated by the two Cp′ ligands, which are closer to dysprosium (average Dy–C=2.64 Å) than phosphorus (average Dy–P=2.934 Å), consistent with the electronic properties of closely related metallocene-based SMMs^34^,^35^,^36^,^37^. The very small angles between the anisotropy axes in the ground and first-excited Kramers' doublets in **2-Dy**, combined with their strong magnetic axiality (Table 2 and Fig. 3), are necessary conditions for magnetic relaxation via the second-excited doublet states at higher temperatures^6^,^14^,^38^. The calculated energy of the second-excited state (Table 1) gives a reasonable agreement with experiment, being higher than the extracted *U*_eff_ value by about 20–40 cm^−1^. Whereas lanthanide SMMs can be designed with ligand environments that promote strongly axial magnetic ground states, targeting SMMs with very large *U*_eff_ values also requires the excited states to possess main magnetic axes that lie close to co-linear with the ground state, which is challenging. In the case of **2-Dy**, the electronic and molecular structures of the individual {Cp′_2_Dy{*μ*-P(H)Mes} units appear to fit the requirements for a dysprosium SMM in which the ground |*m*_J_|=15/2 states and |*m*_J_|=13/2 excited states are indeed co-linear. The strong magnetic axiality of the dysprosium sites is expected to be preserved in the diluted compound **Y**_**2**_**Dy**, which explains the detection of magnetic hysteresis in this system (Fig. 2).

The dysprosium sites in **3-Dy** display a much smaller total splitting of the ground *J*=15/2 manifold compared with **2-Dy** (Table 1). The different splittings are presumably influenced by subtle differences in the structures of the two complexes, that is, greater distances to the Cp′ ligands (by 0.03–0.09 Å) in **3-Dy**, reduced Cp_cent_-Dy-Cp_cent_ angles (by ∼3.4°) and shorter Dy–P bonds (by 0.10–0.16 Å) together with their greater formal charge. Indeed, the calculated charges on the phosphinidene donor atoms in **3-Dy** range from −0.6 to −0.9, compared with the calculated charges of −0.1 to −0.3 on the phosphide donors in **2-Dy**^39^.

The closely located phosphinidene ligands and their charge produce a much stronger equatorial ligand field, which counteracts the axial field arising from the Cp′ ligands. Although the ground Kramers' doublet is still characterized by |*m*_J_|=15/2, the enhanced equatorial ligand field stabilizes other *m*_J_ states and enables greater mixing in **3-Dy** relative to **2-Dy**. This is reflected in weaker magnetic axiality of the ground state in **3-Dy**. Moreover, the first-excited doublets of **3-Dy** feature relatively large transverse *g*-values (*g*_x_ and *g*_y_ in Table 2), which, combined with the large angles with the ground-state main magnetic axis, leads to fast magnetic relaxation through this doublet at high temperatures.

The *ab initio* results for the individual metal sites were used to compute the exchange spectrum and the magnetic properties of the trimetallic complexes using the POLY_ANISO program^40^,^41^. The fitted Lines exchange parameters are given in Supplementary Table 10. The macroscopic magnetic properties were further computed on the basis of the exchange spectrum. Given the strong magnetic axiality of the ground-state Kramers' doublets on the dysprosium sites, the magnetic interaction (exchange+dipolar) between them can be described by the non-collinear Ising Hamiltonian in equation 1.


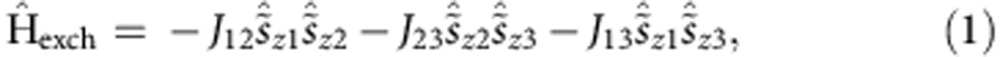


**Figure 4:**
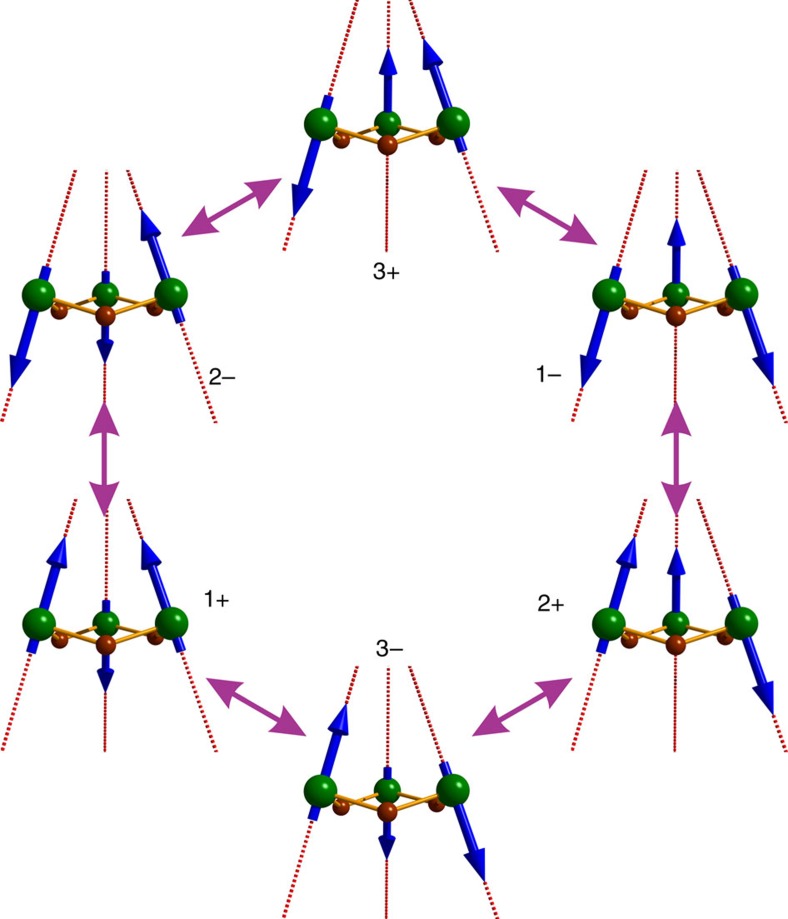
The three low-lying quasidegenerate exchange levels of 2-Dy. The six magnetic eigenstates are related via flips of magnetic moments (blue arrows) on the individual dysprosium sites. The + and − symbols represent time-reversal components corresponding to one exchange Kramers' doublet.

In equation 1, 
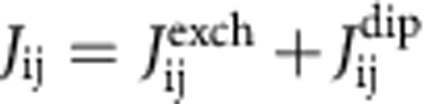
 is the parameter of the total magnetic interaction between metal sites *i* and *j*, including the dipolar and exchange contributions (Supplementary Table 10), 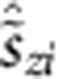 is the projection of the pseudo-spin ½, describing the ground Kramers' doublet on the *i*-th dysprosium site, on the corresponding main magnetic axis *z*_i_. The exchange and magnetic dipolar interactions between dysprosium sites in both compounds are antiferromagnetic. For the calculated directions of the main anisotropy axes on the dysprosium sites in **2-Dy** and **3-Dy** (Fig. 3), the antiferromagnetic exchange and dipolar interactions will stabilize the sixfold degenerate (that is, three Kramers' doublets), frustrated exchange Kramers' doublets (Supplementary Table 10). In a frustrated manifold, magnetization blocking of the exchange type is suppressed because all its states can be accessed by reversal of individual magnetic moments on Dy sites (illustrated for **2-Dy** in Fig. 4)^42^. As a consequence, a quantum tunnelling of the magnetization process as fast as those in single-ion complexes is expected. Under these circumstances, the magnetization blocking is expected to arise from individual dysprosium ions. Actually, the magnetic moment reversal on the individual dysprosium ions in the trimetallic compounds **2-Dy** and **3-Dy** is expected to be faster than in the isostructural complexes containing one single dysprosium, that is, **2-Y**_**2**_**Dy** and **3-Y**_**2**_**Dy**, because in a frustrated compound the presence of nearest neighbour magnetic sites will enhance the magnetization reversal rate on each magnetic ion^42^,^43^. This is the reason for the clearly enhanced blocking of magnetization and the observation of pronounced hysteresis in in **2-Y**_**2**_**Dy** (Fig. 2)^42^.

Magnetization blocking of the exchange type is expected to be efficient only at temperatures not exceeding the exchange splitting, which is the case here because the magnetic coupling constant between two dysprosium ions in **2-Dy** is ∼4 cm^−1^ (Supplementary Table 10). The calculated exchange splitting for **2-Dy** also provides insight into the steps observed in the *M*(*H*) profile for **2-Y**_**2**_**Dy**: the exchange splitting in partially doped **2-YDy**_**2**_ will be about 2 cm^−1^, that is, ∼2,000 Oe, which is in good agreement with the experimentally observed steps at 1,800 Oe.

## Discussion

We have reported a new route to rare-earth complexes of phosphide and phosphinidene ligands. The a.c. susceptibility measurements on phosphide-ligated SMM **2-Dy** reveal a large effective energy barrier of *U*_eff_=210(5) cm^−1^, increasing to 256(6) cm^−1^ on dilution in **2-Y**_**2**_**Dy**. In contrast, **1-Dy** and phosphinidene-ligated [Li(thf)_4_]_2_[**3-Dy**], and their diluted analogues, do not show prominent SMM properties. Theoretical studies of **2-Dy** and **3-Dy** identify that the main magnetic axes on the Dy^3+^ ions in the ground Kramers' doublet have similar orientations approximately perpendicular to the Dy_3_ plane; even allowing for small differences in the axis orientations (of about 10°), the phosphorus atoms occupy equatorial positions, hence the greater electrostatic demands of the phosphinidene ligands diminish the axiality. In **2-Dy**, the thermal magnetic relaxation proceeds via the second-excited Kramers' doublet owing to the near-collinearity of the magnetization axes in the ground- and first-excited doublets. The reduced axiality of the first-excited Kramers' doublet in **3-Dy**, combined with different orientations of the main magnetic axes in the ground- and first-excited doublets, explains the weak magnetic blocking in this system.

The field dependence of the magnetization for **2-Dy** shows very narrow hysteresis at 1.8 K, however butterfly-shaped hysteresis was found up to 4.4 K for **2-Y**_**2**_**Dy**. The properties of undiluted **2-Dy** can be explained in terms of the exchange coupling and frustrated ground state, which enable rapid reversal of the magnetic moments on the individual dysprosium sites. The absence of exchange coupling in **2-Y**_**2**_**Dy** allows more prominent blocking of the magnetization.

Substantial changes to SMM properties can be achieved using a bottom-up approach in which dysprosium phosphide complexes are converted into their phosphinidene analogues. The general features of the chemistry and the electronic structure suggest that exploring other lanthanides and other unconventional ligands in place of phosphorus should enable further enhancements in the SMM behaviour.

## Methods

### General synthesis considerations

All manipulations were performed under an atmosphere of dry, oxygen-free argon, using either standard Schlenk techniques or an argon-filled glove box. Toluene and thf, and their deuterated analogues for NMR spectroscopy, were dried by refluxing over potassium and collected by distillation. All solvents were stored over activated 4 Å molecular sieves and freeze-thaw degassed before use. Anhydrous rare-earth chlorides (99.99% purity) were purchased from Strem Chemicals. *n*-Butyllithium (1.6 M in hexanes), phosphorus(III) chloride, lithium aluminium hydride, mesitylmagnesium bromide (1.0 M in thf) and di-methylcyclopentadiene (90%) were purchased from Sigma-Aldrich. Mesitylphosphine^44^, the sodium salt of methylcyclopentadienide^45^, and rare-earth tris(methylcyclopentadienide) complexes were synthesised according to the literature procedures^46^. Elemental analyses were carried out by Mr Stephen Boyer at London Metropolitan University, UK

### [Cp′_3_Dy(PH_2_Mes)] (**1-Dy**)

A solution of MesPH_2_ in toluene (0.5 M, 4.0 ml, 2.0 mmol) was added to a solution of Cp′_3_Dy (0.80 g, 2.0 mmol) in toluene (4 ml) at room temperature, and the reaction mixture was stirred for 1 h. The resulting pale yellow solution was filtered, concentrated and stored at −30 °C overnight, which resulted in the formation of **1-Dy** as colourless crystals (0.92 g, 83% isolated yield). Elemental analysis, found per percentage (calculated per percentage) for **1-Dy**: C, 58.62 (58.74); H, 6.28 (6.21).

### [Cp′_3_Y(PH_2_Mes)] (**1-Y**)

The synthesis of **1-Y** was accomplished using the procedure described above for **1-Dy**, with the following amounts: MesPH_2_ (0.5 M, 4.0 ml, 2.0 mmol), Cp′_3_Y (0.65 g, 2.0 mmol) and toluene (4 ml). **1-Y** formed as colourless crystals (0.71 g, 75% isolated yield). Elemental analysis, found percentage (calculated percentage) for **1-Y**: C, 67.39 (67.78); H, 7.21 (7.16). ^1^H NMR (benzene-D_6_, 298.15 K, *δ*/p.p.m.): 6.65 (s, 2H, mesityl CH); 5.85, 5.72 (m, 12H, C_5_H_4_CH_3_); 3.86 (d, 2H, PH_2_, ^1^*J*_P–H_=251 Hz); 2.16 (s, 6H, *ortho* CH_3_); 2.10 (s, 9H, Cp′ CH_3_); 2.03 (s, 3H, *para* CH_3_). ^31^P NMR (benzene-D_6_, 298.15 K, *δ*/p.p.m.): −130.80.

### [(Cp′_2_Dy){*μ*-P(H)Mes}]_3_·toluene (**2-Dy**·toluene)

^*n*^BuLi (1.6 M in hexanes, 0.66 ml, 1.06 mmol) was added to a solution of **1-Dy** (0.55 g, 1.06 mmol) in toluene (20 ml) at −78 °C, and the reaction mixture was stirred for 1 h. The reaction was then slowly warmed to room temperature overnight, after which time a pale yellow solution and a precipitate had formed. The resulting pale yellow solution was filtered and concentrated, which resulted in the formation of a yellow precipitate. The precipitate was re-dissolved and the solution stored at −30 °C overnight, which resulted in the formation of **2-Dy·**toluene as colourless crystalline blocks (0.34 g, 63%). Elemental analysis, found percentage (calculated percentage) for **2-Dy·**toluene: C, 55.63 (55.76); H, 5.83 (5.75).

### [(Cp′_2_Y){*μ*-P(H)Mes}]_3_·toluene (**2-Y**·toluene)

The synthesis of **2-Y**·toluene was accomplished using the procedure described above for **2-Dy**·toluene, with the following amounts: **1-Y** (0.59 g, 1.24 mmol), toluene (20 ml) and ^*n*^BuLi (1.6 M in hexanes, 0.77 ml, 1.24 mmol). **2-Y**·toluene was isolated as colourless crystalline blocks (0.37 g, 70% based on yttrium). Elemental analysis, found percentage (calculated percentage) for **2-Y·**toluene: C, 65.16 (65.32); H, 6.82 (6.74). ^1^H NMR (toluene-D_8_, 298.15 K, *δ*/p.p.m.): 6.78–6.93 (s, 6H, mesityl CH); 5.50–6.55 (s, 24H, C_5_H_4_CH_3_); 3.47–1.61 (s, overlapping peaks due to CH_3_ groups). ^31^P NMR (benzene-D_6_, 298.15 K, *δ*/p.p.m.): −124.28, −145.21 and −142.65.

### [Li(thf)_4_]_2_[(Cp′_2_Dy)_3_(μ-PMes)_3_Li]·thf ([Li(thf)_4_]_2_[3-Dy]·thf)

A solution of **2-Dy·**toluene (0.38 g, 0.25 mmol) in thf (10 ml) was cooled to −78 °C and ^*n*^BuLi (1.6 M in hexanes, 0.47 ml, 75 mmol) was added drop wise. After stirring at −78 °C for 30 min, the reaction was warmed to room temperature over 3 h, after which time an orange colour had developed. The solution was concentrated until copious amounts of precipitate had formed, then the precipitate was re-dissolved by gentle heating and the solution stored at +4 °C. [Li(thf)_4_]_2_[**3-Dy**]·thf formed as large orange blocks (0.33 g, 64%). Elemental analysis, found percentage (calculated per %) for [Li(thf)_4_]_2_[**3-Dy**]·thf: C, 56.88 (57.10); H, 7.25 (7.12).

### [Li(thf)_4_]_2_[(Cp′_2_Y)_3_(*μ*-PMes)_3_Li]·thf ([Li(thf)_4_]_2_[3-Y]·thf)

The synthesis of [**3-Y**][Li(thf)_4_]_2_·thf was accomplished using the procedure described above for [**3-Y**][Li(thf)_4_]_2_·thf, with the following amounts: **2-Y·**toluene (0.34 g, 0.27 mmol), thf (10 ml) and ^*n*^BuLi (1.6 M in hexanes, 0.50 ml, 0.79 mmol). [Li(thf)_4_]_2_[**3-Y**]·thf was isolated as pale orange crystalline blocks (0.27 g, 56% based on yttrium). Elemental analysis, found per % (calculated per %) for [**3-Y**][Li(thf)_4_]_2_·thf: C, 63.69 (63.77); H, 8.02 (7.95). NMR spectra were acquired in dimethoxyethane (that is, C_4_H_10_O_2_) with a few drops of benzene-D_6_ for the signal lock. ^1^H (298.15 K, *δ*/p.p.m.): 6.78, 6.68 (2 × s, 2 × 3H, mesityl CH); 6.47, 6.15, 4.99 and 4.74 (s, 4 × 6H, C_5_H_4_CH_3_); 3.46 (m, 4H, thf CH_2_OCH_2_); 2.59 (s, 9H, *ortho* CH_3_); 2.52 (s, 9H, *ortho* CH_3_); 2.26 (s, 9H, Cp′ CH_3_); 2.08 (s, 9H, Cp′ CH_3_); 1.52 (m, 4H, thf CH_2_CH_2_O); 1.46 (s, 9H, *para* CH_3_). ^31^P NMR (benzene-D_6_, 298.15 K, *δ*/p.p.m.): +57.24 p.p.m.; a small amount of MesPH_2_ is observed at −157.51 p.p.m., which occurs due to the phosphinidene ligand reacting slowly with the DME solvent.

### Doped sample (**2-Y**_**2**_ **Dy**)/(**2-Y**)

The dilution experiment to obtain **2-Y**_**2**_**Dy** doped into a matrix of **2-Y** was achieved by combining **1-Dy** (0.0138, g, 0.025 mmol) and **1-Y** (0.2280, g, 0.475 mmol; that is, a 5:95 stoichiometric ratio) as solids, and dissolving the mixture in toluene (8 ml). Following the addition of ^*n*^BuLi (1.6 M, 0.31 ml, 0.50 mmol) at −78 °C and a workup procedure as described for **2-Dy**, the doped sample was obtained as colourless crystals (0.088 g, 41% based on metal content).

### Doped sample [Li(thf)_4_]_2_[3-Y_2_Dy]·thf/[Li(thf)_4_]_2_[3-Y]·thf

The dilution experiment to obtain [Li(thf)_4_]_2_[**3-Y**_**2**_**Dy**]·thf doped into a matrix of [Li(thf)_4_]_2_[**3-Y**]·thf was achieved by the deprotonation of **2-Y**_**2**_**Dy** (0.14 g, 0.11 mmol) by of ^*n*^BuLi (1.6 M, 0.20 ml, 0.32 mmol) at −78 °C, with the workup procedure as described for [Li(thf)_4_]_2_[**3-Dy**]·thf. The doped sample was obtained as pale orange crystals (0.043 g, 22% based on metal content).

### Characterization of doped materials

The two doped materials were characterized by X-ray diffraction (Supplementary Table 1): measurements of the unit cell parameters of several crystals of each were performed on an Oxford Xcaliber-2 diffractometer using Mo-K*α* radiation at 100 K. The unit cell dimensions of both compounds were found to be equivalent (within 3*σ*) to those observed for their respective pure yttrium compounds **2-Y** and [Li(thf)_4_]_2_[**3-Y**]·thf. Accurate dysprosium/yttrium ratios were measured by inductively coupled plasma atomic emission spectroscopy using a Thermo iCap 6300 ICP-OES instrument, which resulted in dysprosium contents of 5.0±0.5% for both doped materials.

### Magnetic property measurements

The magnetic properties were measured using a Quantum Design MPMS-7 superconducting quantum interference device magnetometer at temperatures in the range of 1.8–300 K. In a glove box, polycrystalline samples of each material were transferred to NMR tubes and restrained in eicosane. The NMR tubes were then placed under a partial vacuum and flame sealed before being transferred to the magnetometer.

### Computational details

All calculations were done with MOLCAS 7.8 and are of CASSCF/RASSI/SINGLE_ANISO type. For each of **1-Dy**, **2-Dy** and **3-Dy**, two computational models were employed. In model A, all the CH_3_ groups were replaced by hydrogen atoms. Model B is the entire molecule as determined by X-ray crystallography, using all the atomic coordinates in the CIF files. The structures of model A (small) for **1-Dy**, **2-Dy** and **3-Dy** are shown in Supplementary Figs 21–23, respectively. Two basis set approximations were used: **1**—small (DZP quality), and **2**—large (TZP quality). We have therefore considered four computational models for each magnetic centre in the investigated molecules: A1, A2, B1 and B2. The following results refer to the most complete model B2 (full structure with large basis set). Details of the calculations for models A1, A2 and B1 can be obtained from LFC. Each magnetic centre was computed, and neighbouring metal ions were computationally replaced by diamagnetic Lu^3+^. The active space of the CASSCF method includes the electrons from the last shell spanning the seven 4f orbitals of the Ln^3+^ ion. For all calculations, it was possible to mix only a limited number of roots, namely 21 sextets, 128 quartet and 130 doublet states. On the basis of the resulting spin-orbital multiplets, the SINGLE_ANISO programme computed the local magnetic properties (*g* tensors, main magnetic axes, local magnetic susceptibility, crystal-field parameters and so on). The *ab initio* results for the individual metal sites were used to compute the exchange spectrum and the magnetic properties of the trimetallic complexes using the POLY_ANISO program. The exchange interaction between the dysprosium sites is considered within the Lines model, while the contribution of the intramolecular dipole–dipole magnetic coupling is accounted for exactly given that all necessary data are available from the *ab initio* calculations.

All three exchange interactions in **1-Dy** and **2-Dy** were described by one single Lines exchange parameter. The values of the Lines parameter for **1-Dy** and **2-Dy** was determined by minimizing the difference between the calculated and the measured magnetic susceptibility. The Lines parameters were expressed in terms of the Ising Hamiltonian by the expression 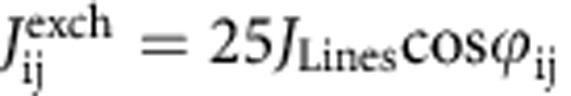, where *ϕ*_ij_ is the angle between the main anisotropy axes (*z*_i_ and *z*_j_) of the interacting sites. Supplementary Fig. 25 shows the comparison of the calculated and measured magnetic susceptibility.

## Additional information

**Accession codes.** The X-ray crystallographic coordinates for the structures reported in this Article have been deposited at the Cambridge Crystallographic Data Centre (CCDC), under deposition numbers 1032227-1032232. These data can be obtained free of charge from the Cambridge Crystallographic Data Centre via www.ccdc.cam.ac.uk/data_request.cif.

**How to cite this article:** Pugh, T. *et al.* Influencing the properties of dysprosium single-molecule magnets with phosphorus donor ligands. *Nat. Commun.* 6:7492 doi: 10.1038/ncomms8492 (2015).

## Supplementary Material

Supplementary Figures and Supplementary Tables Supplementary Figures 1-26 and Supplementary Tables 1-10

Supplementary Data 1 cif file for 1-Dy

Supplementary Data 2 cif file for 2-Dy.toluene

Supplementary Data 3 cif file for [Li(thf)4]2[3-Dy].thf

Supplementary Data 4 cif file for 1-Y

Supplementary Data 5 cif file for 2-Y.toluene

Supplementary Data 6 cif file for [Li(thf)4]2[3-Y].thf

## Figures and Tables

**Table 1:** Calculated low-lying energy (cm^−1^) spectrum of individual Dy^3+^ sites in **1-Dy**, **2-Dy** and **3-Dy**.

**KD**	**1-Dy**	**2-Dy**			**3-Dy**		
		**Dy1**	**Dy2**	**Dy3**	**Dy1**	**Dy2**	**Dy3**
1	0.0	0.0	0.0	0.0	0.0	0.0	0.0
2	45.2	126.7	134.2	135.0	63.8	69.5	100.7
3	98.7	277.3	296.8	297.8	77.4	88.8	104.4
4	287.1	345.8	320.9	334.4	116.2	108.5	132.9
5	339.5	362.7	388.7	381.0	136.0	148.5	150.2
6	391.7	402.8	431.0	419.0	160.6	160.9	181.1
7	452.5	433.6	499.2	478.0	194.0	187.1	217.2
8	583.9	551.7	675.6	637.8	232.1	271.2	290.6

KD, Kramers' doublet.

**Table 2:** Calculated *g* tensors of the ground- and first-excited Kramers' doublets and the angles between their main anisotropy axes in **1-Dy**, **2-Dy** and **3-Dy**.

							
