# Supplementary material for: Influencing the properties of dysprosium single-molecule magnets with phosphorus donor ligands
Source: Nat Commun. 2015 Jul 1;6:7492. doi: 10.1038/ncomms8492 (PMC4507012; doi:10.1038/ncomms8492)
Supplement: Supplementary Figures and Supplementary Tables — Supplementary Figures 1-26 and Supplementary Tables 1-10 [file ncomms8492-s1.pdf]

Supplementary Figures

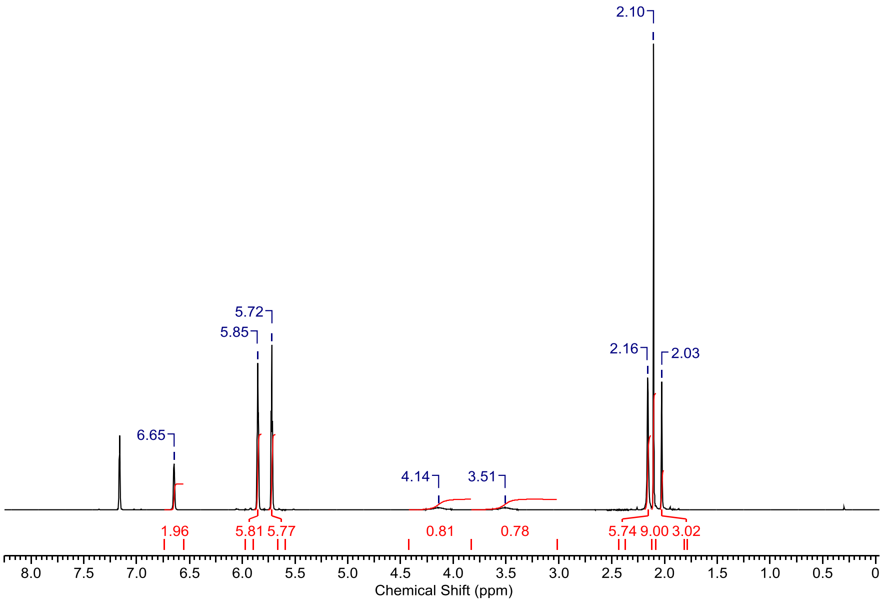

Supplementary Figure 1. ^{1}H NMR spectrum of **1-Y** in benzene-D$_6$ at 298 K.

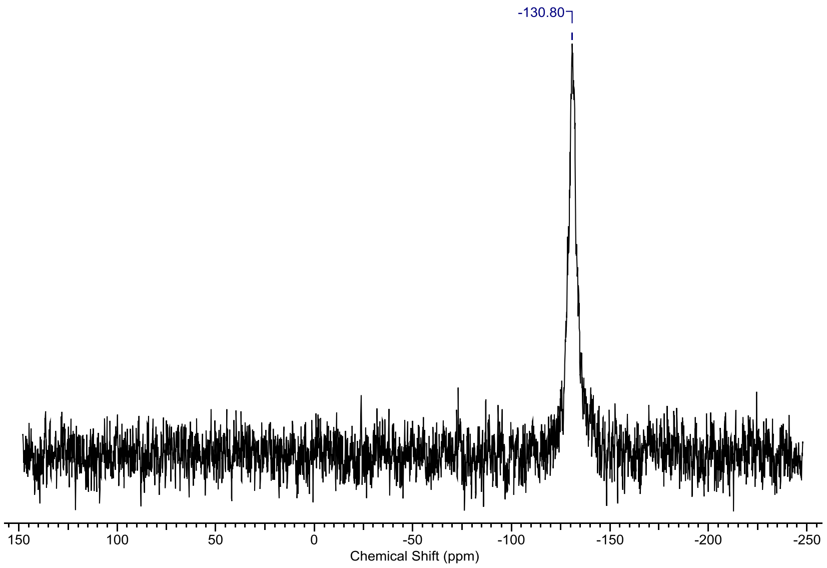

Supplementary Figure 2. ^{31}P NMR spectrum of **1-Y** in benzene-D$_6$ at 298 K.

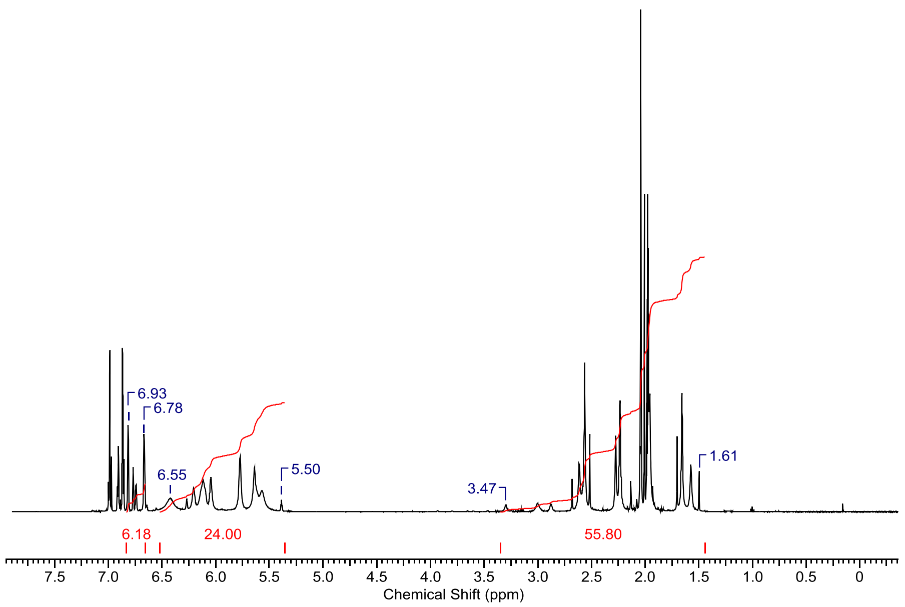

Supplementary Figure 3. ^{1}H NMR spectrum of **2-Y**·toluene in toluene-D_8 at 298 K. The integral for the region 1.61-3.47 ppm includes residual protons in the solvent, which overlap with the resonances due to **2-Y**.

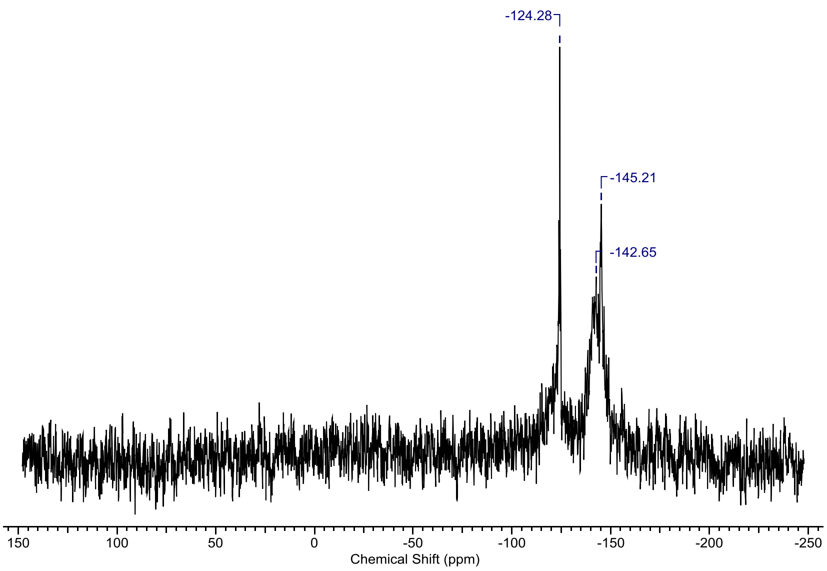

Supplementary Figure 4. $^{31}P\{^{1}H\}$ NMR spectrum of **2-Y**·toluene in toluene-D_8 at 298 K.

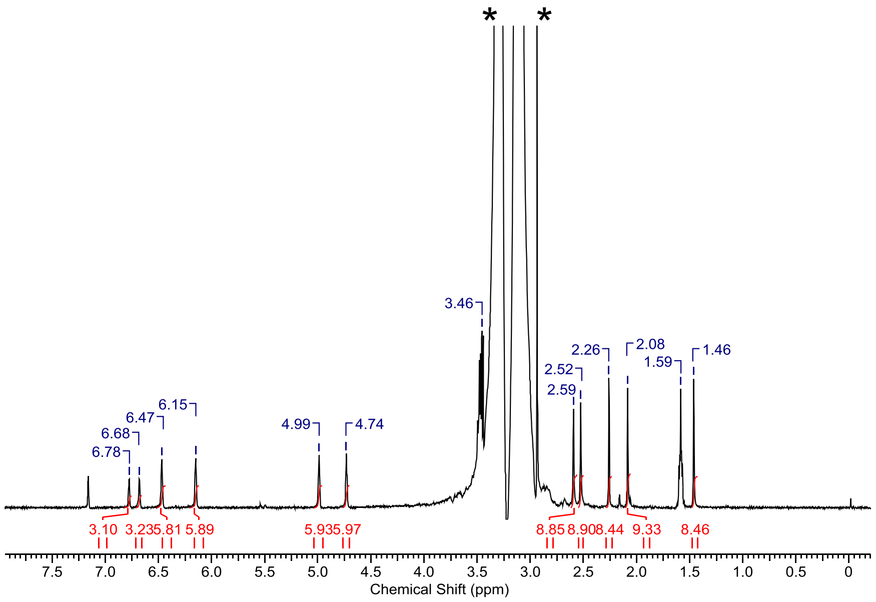

Supplementary Figure 5. ^{1}H NMR spectrum of [**3-Y**][Li(thf)$_4$]$_2$·thf recorded in a mixture of dimethoxyethane ($C_4H_{10}O_2$) and two drops of benzene-D$_6$ at 298 K in thf-D$_8$ at 298 K.

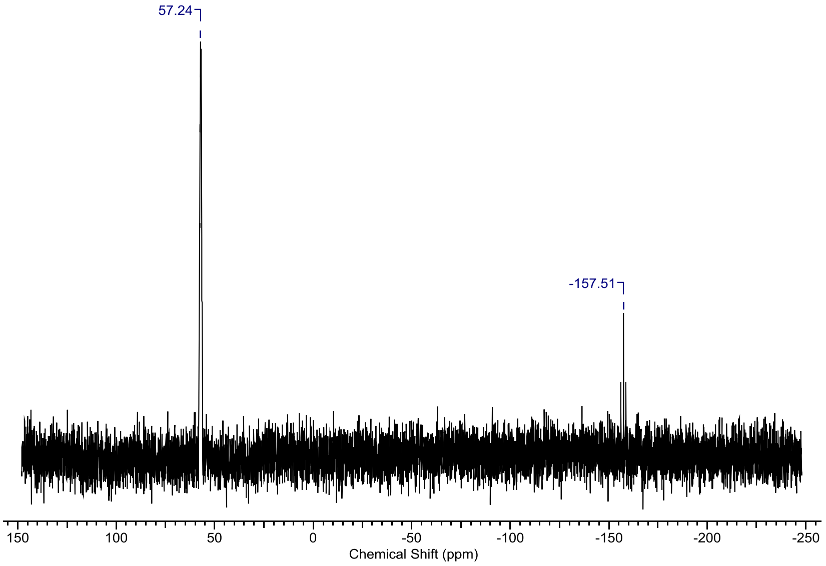

Supplementary Figure 6. ^{1}H-coupled ^{31}P NMR spectrum of [**3-Y**][Li(thf)$_4$]$_2$·thf recorded in a mixture of dimethoxyethane ($C_4H_{10}O_2$) and two drops of benzene-D$_6$ at 298 K in thf-D$_8$ at 298 K.

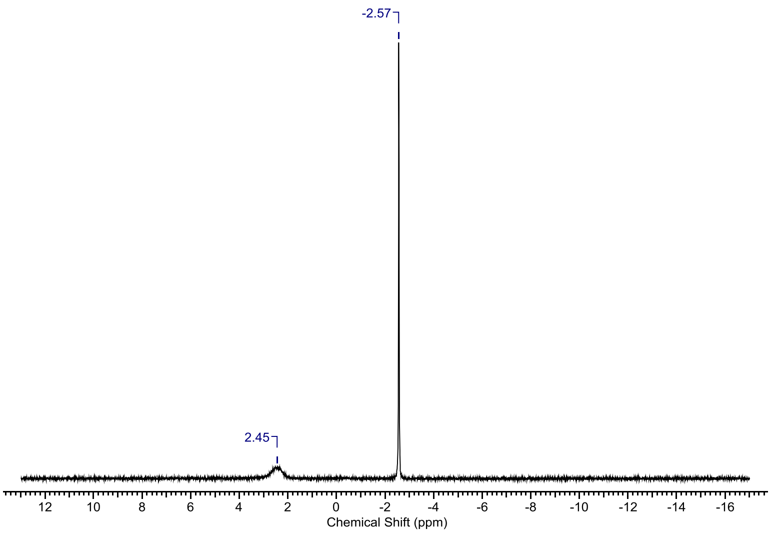

Supplementary Figure 7. ^{7}Li NMR spectrum of [**3-Y**][Li(thf)$_4$]$_2$·thf recorded in a mixture of dimethoxyethane ($C_4H_{10}O_2$) and two drops of benzene-D_6 at 298 K in thf-D_8 at 298 K..

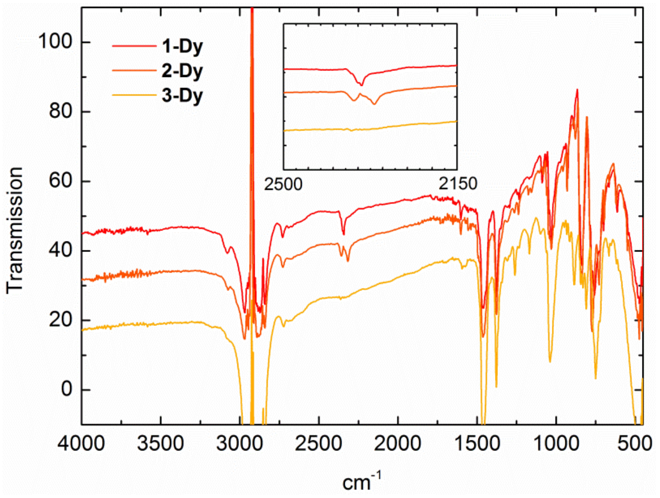

Supplementary Figure 8. Infrared spectra (Nujol mulls) of **1-Dy** ($\tilde{\nu}_{P\text{–}H}$ = 2342 cm^{-1}), **2-Dy**·toluene ($\tilde{\nu}_{P\text{–}H}$ = 2318, 2358 cm^{-1}) and [Li(thf)$_4$]$_2$[**3-Dy**]·thf.

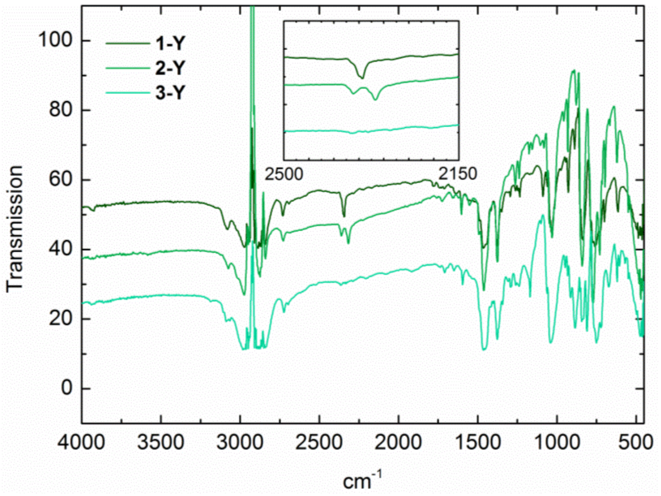

Supplementary Figure 9. Infrared spectra (Nujol mulls) of **1-Y** ($\tilde{\nu}_{P-H}$ = 2344 cm^{-1}), **2-Y**·toluene ($\tilde{\nu}_{P-H}$ = 2316, 2362 cm^{-1}) and [Li(thf)$_4$]$_2$[**3-Y**]·thf.

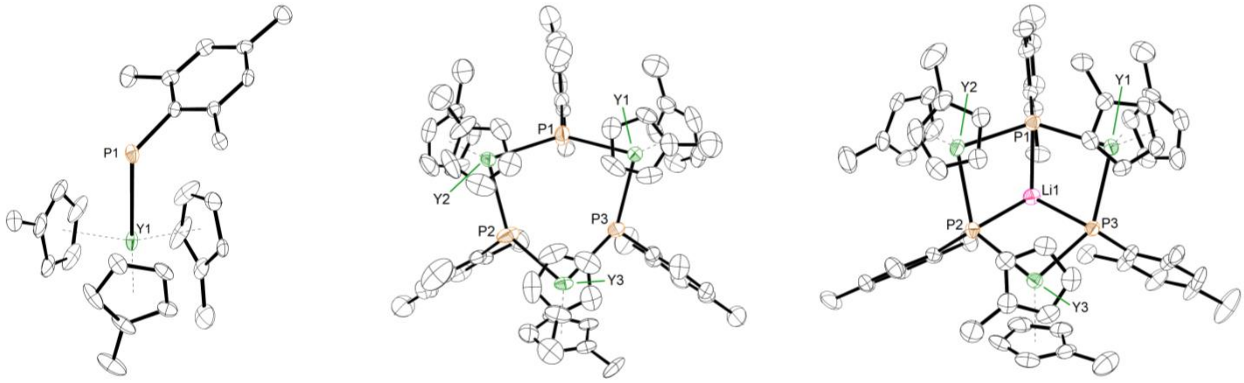

Supplementary Figure 10. Thermal ellipsoid representations (50% probability) of the molecular structures of **1-Y** (left), **2-Y** (centre) and **3-Y** (right). For clarity, hydrogens atoms are omitted from **1-Y** and **2-Y**.

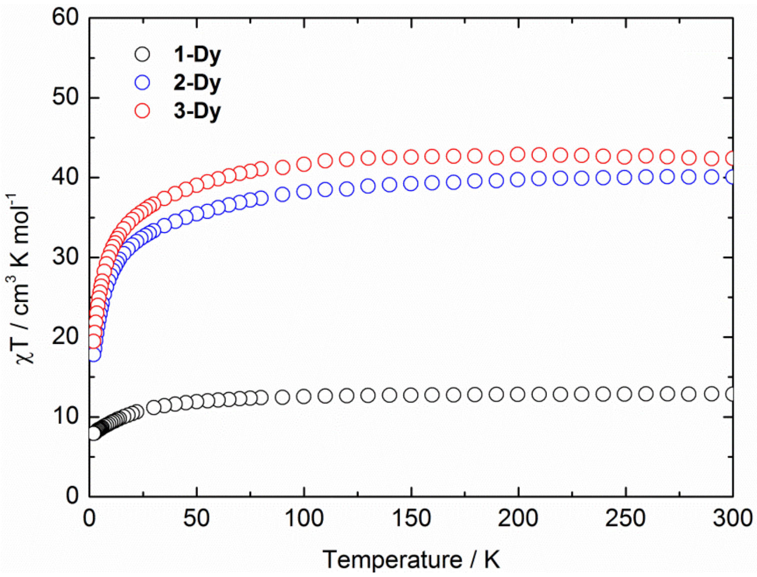

Supplementary Figure 11. Temperature dependence of the product of the molar magnetic susceptibility (χ_M) with temperature for **1-Dy**, **2-Dy**·toluene, [**3-Dy**][Li(thf)$_4$]$_2$·thf.

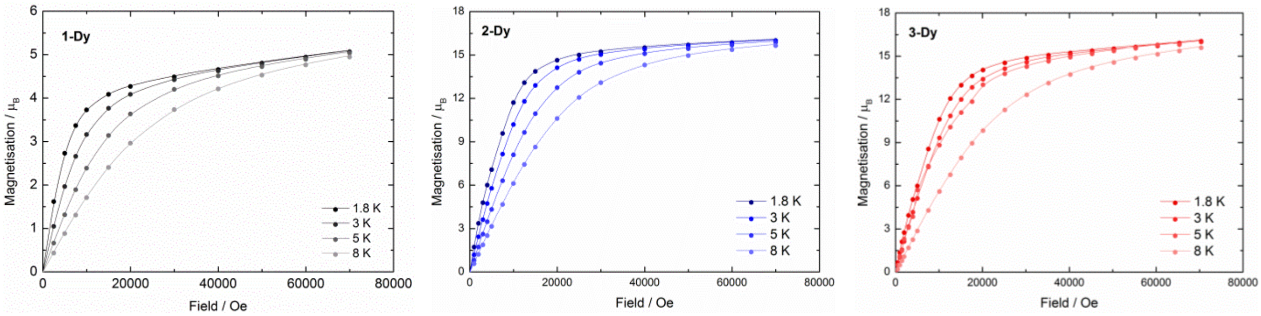

Supplementary Figure 12. Field dependence of the magnetization for **1-Dy** (left), **2-Dy**·toluene (centre) and [**3-Dy**][Li(thf)$_4$]$_2$·thf (right) at temperatures in the range 1.8-8 K.

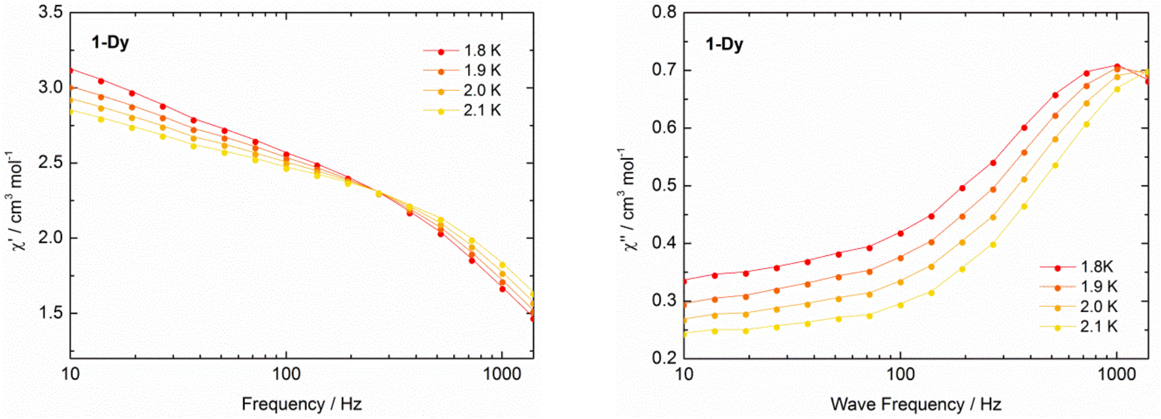

Supplementary Figure 13. Frequency dependence of the in-phase (χ') and the out-of-phase (χ'') magnetic susceptibility for **1-Dy** using an oscillating field of H_{ac} = 1.55 Oe and zero applied field.

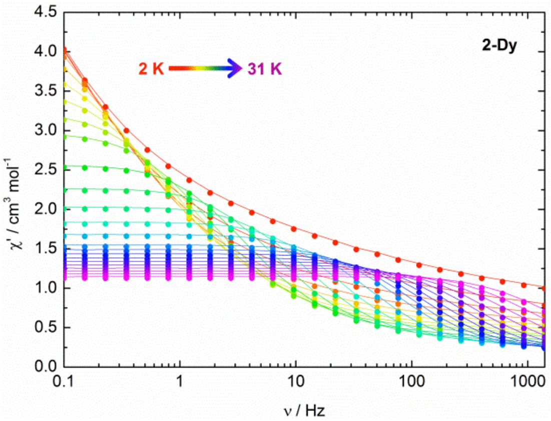

Supplementary Figure 14. Frequency dependence of the in-phase (χ') magnetic susceptibility for **2-Dy** using an oscillating field of H_{ac} = 1.55 Oe and zero applied field.

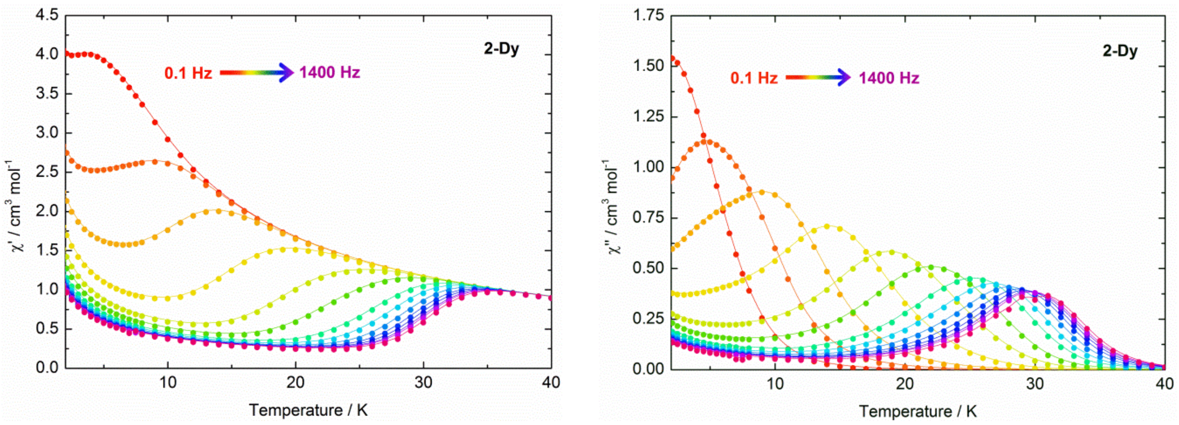

Supplementary Figure 15. Temperature dependence of the in-phase (χ') and the out-of-phase (χ'') magnetic susceptibility for **2-Dy**, using an oscillating field of H_{ac} = 1.55 Oe and zero applied field.

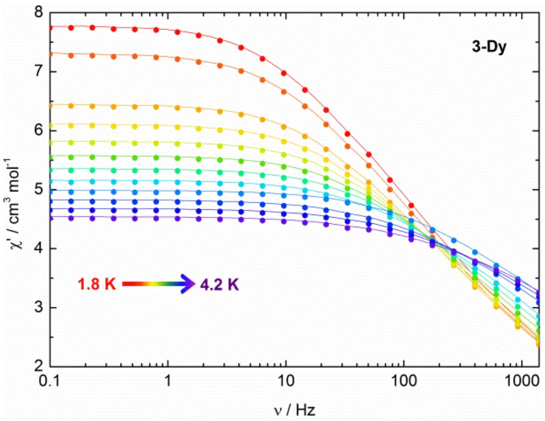

Supplementary Figure 16. Frequency dependence of the in-phase (χ') magnetic susceptibility for [**3-Dy**]$^-$, using an oscillating field of H_{ac} = 1.55 Oe and zero applied field.

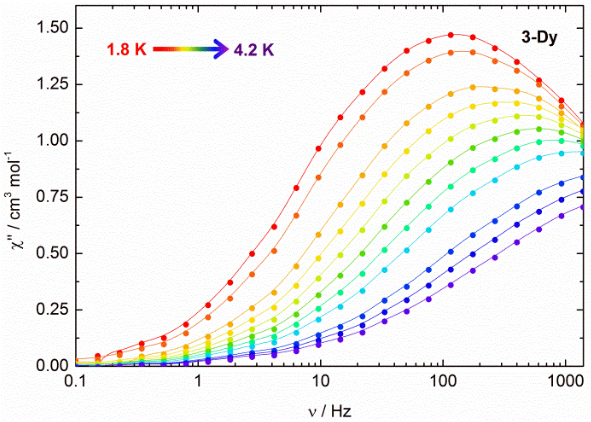

Supplementary Figure 17. Frequency dependence of the out-of-phase (χ'') magnetic susceptibility for [**3-Dy**]$^-$, using an oscillating field of H_{ac} = 1.55 Oe and zero applied field.

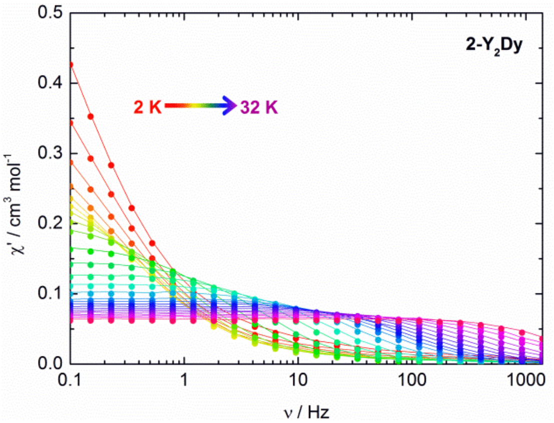

Supplementary Figure 18. Frequency dependence of the in-phase (χ')magnetic susceptibility for **2-Y$_2$Dy** in a matrix of **2-Y** (1:20 Dy:Y). Data collected using an oscillating field of H_{ac} = 1.55 Oe and zero applied field.

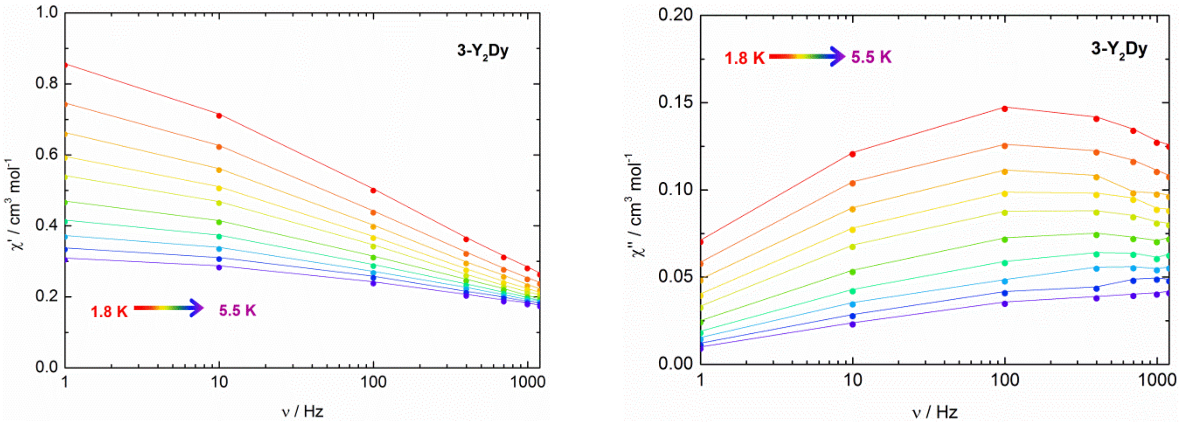

Supplementary Figure 19. Frequency dependence of the in-phase (χ') and the out-of-phase (χ'') magnetic susceptibility for [**3-Y$_2$Dy**][Li(thf)$_4$]$_2$ in a matrix of [**3-Y**][Li(thf)$_4$]$_2$ (1:20 Dy:Y). Data collected using an oscillating field of H_{ac} = 1.55 Oe and zero applied field.

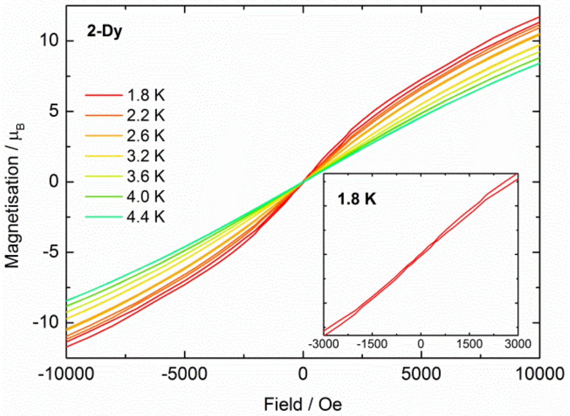

Supplementary Figure 20. Field (H) dependence of the magnetization (M) for undiluted **2-Dy** at T = 1.8 K with H = ±5 T. Inset: expansion of the region with H = ±3000 Oe showing the small opening of the hysteresis loop.

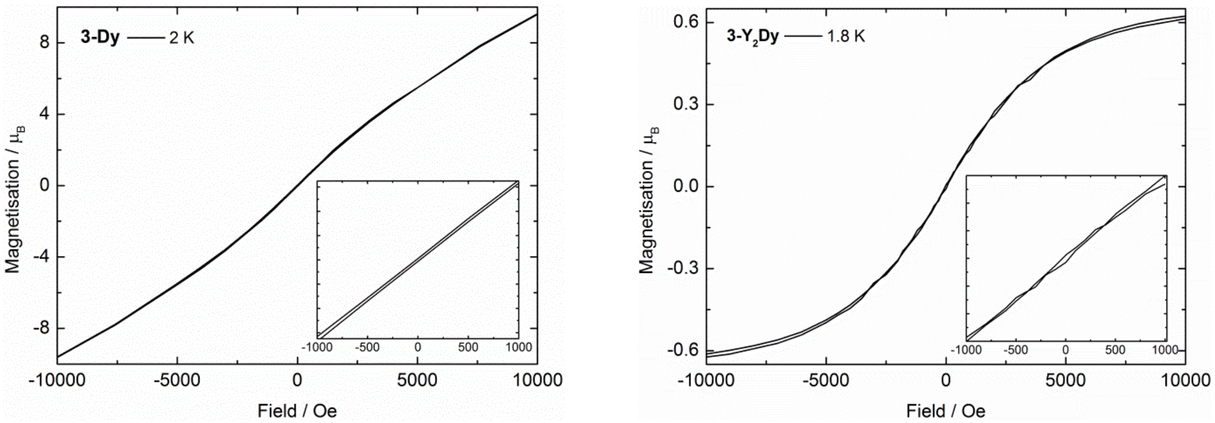

Supplementary Figure 21. Field (*H*) dependence of the magnetization (*M*) for undiluted **3-Dy** (left) and diluted **3-Y_2Dy** (right) at T = 1.8 K and 2 K, respectively, with $H = \pm 5$ T. Inset: expansion of the region with $H = \pm 1000$ Oe showing the small opening of the hysteresis loop.

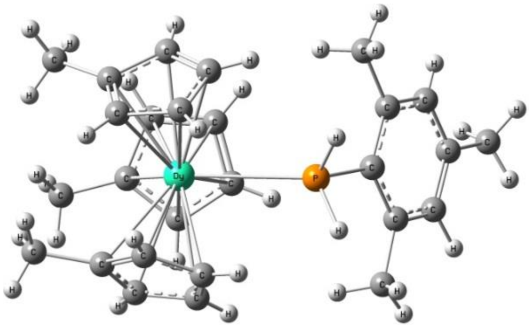

Supplementary Figure 22. Calculated structure of **1-Dy**.

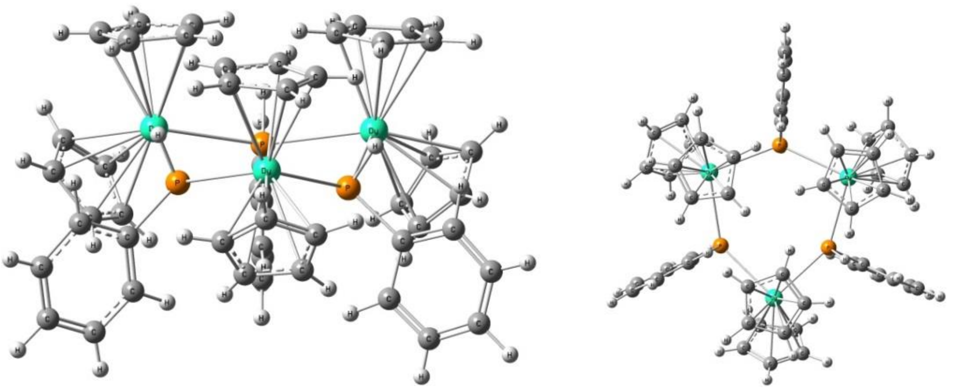

Supplementary Figure 23. Calculated structure of **2-Dy**.

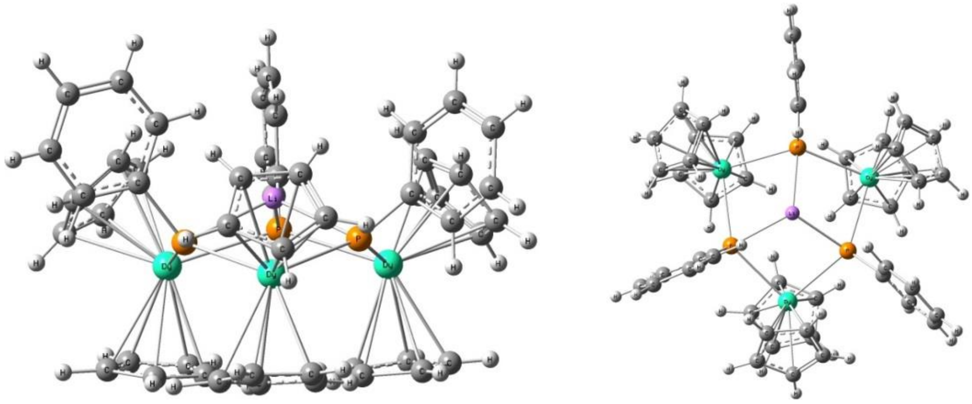

Supplementary Figure 24. Calculated structure of **3-Dy**.

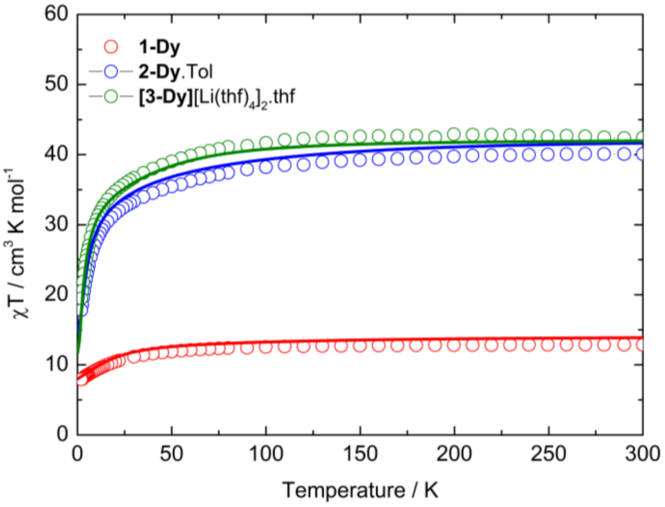

Supplementary Figure 25. Calculated temperature dependence of the product of the molar magnetic susceptibility (χ_M) with temperature (solid lines) for **1-Dy**, **2-Dy**·toluene, [**3-Dy**][Li(thf)$_4$]$_2$·thf compared to the experimental data (circles).

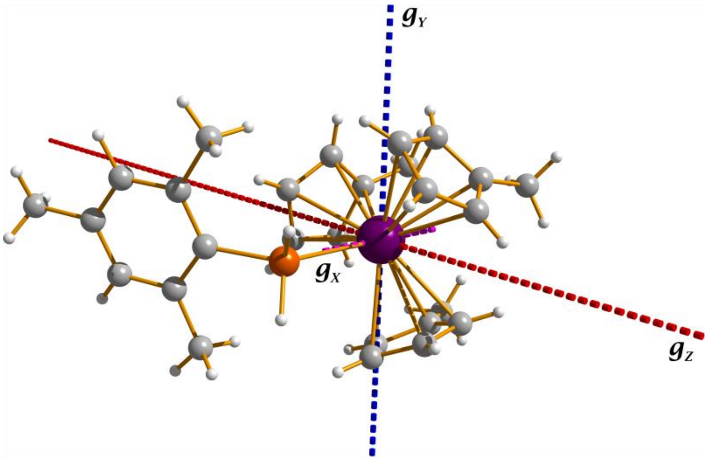

Supplementary Figure 26. Orientation of the magnetic axes in the ground Kramers' doublets for **1-Dy**. The Dy–P bond and the g_z axis create an angle of 88.4°.

Supplementary Tables

Supplementary Table 1. Crystal data and structure refinement details. MoKα (λ = 0.71073)

	**1-Dy**	**2-Dy·toluene**	**[3-Dy][Li(thf)$_4$]$_2$·thf**	**1-Y**	**2-Y·toluene**	**[3-Y][Li(thf)$_4$]$_2$·thf**
empirical formula	$C_{27}H_{34}DyP$	$C_{70}H_{83}Dy_3P_3$	$Dy_3P_3C_{99}H_{132}O_9Li_3$	$C_{27}H_{34}YP$	$C_{70}H_{83}Y_3P_3$	$Y_3P_3C_{99}H_{147}O_9Li_3$
formula weight	552.01	1504.77	2067.27	478.42	1284.00	1861.62
temperature / K	100(2)	150(1)	150(1)	100(2)	150(2)	150(1)
crystal system	monoclinic	monoclinic	monoclinic	monoclinic	monoclinic	monoclinic
space group	$P2_1/n$	Cc	$P2_1/c$	$P2_1/n$	$P2_1/c$	$P2_1/c$
a / Å	8.1970(5)	14.8953(6)	22.3210(5)	8.1790(6)	11.6328(4)	22.3184(10)
b / Å	24.7447(18)	18.6307(8)	15.7702(3)	24.6788(18)	25.0914(10)	15.7523(10)
c / Å	11.5021(10)	23.6059(9)	27.9921(7)	11.5031(10)	21.5705(7)	27.9702(14)
α / °	90	90	90	90	90	90
β / °	94.741(7)	103.260(4)	95.319(2)	94.707(6)	91.002(3)	91.358(4)
γ / °	90	90	90	90	90	90
volume / Å^3	2325.0(3)	6376.3(5)	9811.0(4)	2314.0(3)	6295.1(4)	9790.4(9)
Z	4	4	4	4	4	4
ρ_{calc} / mg mm^{-3}	1.577	1.568	1.400	1.373	1.355	1.263
crystal size / mm^3	0.5 × 0.3 × 0.2	0.4 × 0.2 × 0.15	0.5 × 0.3 × 0.2	0.2 × 0.1 × 0.05	0.1 × 0.05 × 0.02	0.5 × 0.1 × 0.02
2θ range/°	5.88-52.744	6.888-52.726	6.288-50.7	5.89-52.742	7.46-52.746	6.512-50.7
reflections collected	9055	23340	29590	9217	37554	37632
independent reflections	4745	9579	17805	4724	12814	17853
R(int)	0.0419	0.0737	0.0331	0.0645	0.0456	0.0797
data/restraints/parameters	4745/68/316	9579/131/701	17805/18/1107	4724/0/286	12814/178/729	17853/333/1275
goodness-of-fit on F^2	1.039	1.064	1.037	1.028	1.039	0.992
final R indexes [I≥2σ(I)]	R_1 = 0.0382<br>wR_2 = 0.0841	R_1 = 0.0570<br>wR_2 = 0.1221	R_1 = 0.0429<br>wR_2 = 0.0917	R_1 = 0.0529<br>wR_2 = 0.1082	R_1 = 0.0570<br>wR_2 = 0.1423	R_1 = 0.0607<br>wR_2 = 0.0881
final R indexes [all data]	R_1 = 0.0487<br>wR_2 = 0.0932	R_1 = 0.0660<br>wR_2 = 0.1307	R_1 = 0.0589<br>wR_2 = 0.1011	R_1 = 0.0971<br>wR_2 = 0.1415	R_1 = 0.0937<br>wR_2 = 0.1604	R_1 = 0.1366<br>wR_2 = 0.1080
largest diff. peak, hole /e.A^{-3}	1.81, –1.83	3.39, –1.05	1.44, –1.12	0.76, –0.91	0.83, –2.03	0.41, –0.41

Supplementary Table 2. Selected bond lengths (Å) and angles (°) for **1-Dy**, **2-Dy** and **3-Dy**

	**1-Dy**	**2-Dy**	**3-Dy**
Dy–P	3.009(1)	2.926(6)-2.951(6)	2.7850(15)-2.8249(15)
Dy–C	2.676(7)-2.744(6)	2.60(2)-2.72(1)	2.625(6)-2.762(6)
Dy–Cp_{cent}	2.420(5)-2.443(5)	2.345(8)-2.369(7)	2.385(3)-2.441(3)
Li–P			2.472(9)-2.557(9)
P-Dy-P		89.23(18)-98.49(18)	90.95(4)-93.99(4)
Cp_{cent}-Dy-Cp_{cent}	117.73(10)-118.56(10)	126.0(3)-126.4(3)	121.23(10)-123.96(10)
Dy-P-Dy		128.65(17)-134.35(18)	131.08(5)-134.99(5)
P-Li-P			107.0(3)-109.6(4)
Dy-P-Li			76.9(2)-80.5(2)

Supplementary Table 3. Selected bond lengths (Å) and angles (°) for **1-Y**, **2-Y** and **3-Y**

	**1-Y**	**2-Y**	**3-Y**
Y–P	3.010(1)	2.923(2)-2.954(2)	2.7869(12)-2.8269(13)
Y–C	2.661(6)-2.749(5)	2.614(7)-2.67(1)	2.633(4)-2.760(6)
Y–Cp_{cent}	2.416(5)-2.444(5)	2.337(7)-2.362(7)	2.379(2)-2.430(2)
Li–P			2.468(8)-2.546(8)
P-Y-P		86.33(5)-91.23(5)	91.03(4)-94.06(4)
Y-P-Y		135.48(7)-137.74(6)	131.29(5)-135.27(5)
P-Li-P			106.8(3)-109.4(3)
Y-P-Li			77.32(18)-79.99(19)

Supplementary Table 4. Energies of the low-lying Kramers doublets for **1-Dy**.

Kramers Doublet	Energy / cm^{-1}
1	0.000
	0.000
2	45.212
	45.212
3	98.749
	98.749
4	287.170
	287.170
5	339.555
	339.555
6	391.739
	391.739
7	452.533
	452.533
8	583.953
	583.953

Supplementary Table 5. *g*-Tensors for the low-lying Kramers doublets for Dy^{3+} in **1-Dy**.

Kramers doublet		Dy1
1	g_x	0.88625
	g_y	5.41752
	g_z	15.01699
2	g_x	2.59868
	g_y	3.35940
	g_z	6.35830
3	g_x	10.17715
	g_y	6.96903
	g_z	2.52102
4	g_x	5.20707
	g_y	7.16596
	g_z	9.70857
5	g_x	1.07105
	g_y	1.56443
	g_z	10.88776
6	g_x	1.26191
	g_y	1.89979
	g_z	13.67944
7	g_x	1.71010
	g_y	1.79953
	g_z	16.47939
8	g_x	0.04281
	g_y	0.04719
	g_z	19.67566

Supplementary Table 6. Energies of the low-lying Kramers doublets for **2-Dy**.

Kramers doublet	Energy / cm^{-1}		
	Dy1	Dy2	Dy3
1	0.000	0.000	0.000
	0.000	0.000	0.000
2	126.738	134.260	135.048
	126.738	134.260	135.048
3	277.317	296.823	297.883
	277.317	296.823	297.883
4	345.800	320.995	334.413
	345.800	320.995	334.413
5	362.707	388.786	381.074
	362.707	388.786	381.074
6	402.858	431.024	419.049
	402.858	431.024	419.049
7	433.617	499.247	478.016
	433.617	499.247	478.016
8	551.778	675.677	637.826
	551.778	675.677	637.826

Supplementary Table 7. *g*-Tensors for the low-lying Kramers doublets for Dy^{3+} in **2-Dy**.

Kramers doublet		Dy1	Dy2	Dy3
1	g_x	0.00033	0.00196	0.00029
	g_y	0.00047	0.00367	0.00050
	g_z	19.43021	19.30007	19.45874
2	g_x	0.00115	0.02129	0.00306
	g_y	0.00155	0.02269	0.00324
	g_z	17.04631	16.85113	16.96575
3	g_x	0.02891	1.06941	0.14877
	g_y	0.03023	2.62004	0.23083
	g_z	14.88347	13.21101	14.80977
4	g_x	10.05515	1.22690	0.11165
	g_y	5.86141	1.91332	0.68495
	g_z	1.63782	14.44525	19.14349
5	g_x	10.10938	3.31114	2.64013
	g_y	5.26252	4.49971	3.31519
	g_z	0.20865	9.74614	10.71650
6	g_x	3.47925	3.45545	3.74371
	g_y	5.70108	5.58090	6.68520
	g_z	9.91449	11.17103	10.54301
7	g_x	1.19063	0.62235	0.55815
	g_y	2.77104	1.04659	1.13718
	g_z	14.49222	16.60486	16.44954

Supplementary Table 8. Energies of the low-lying Kramers doublets for **3-Dy**.

Kramers doublet	Energy / cm^{-1}		
	Dy1	Dy2	Dy3
1	0.000	0.000	0.000
	0.000	0.000	0.000
2	58.247	64.365	95.241
	58.247	64.365	95.241
3	78.371	78.250	104.321
	78.371	78.250	104.321
4	103.435	97.406	120.807
	103.435	97.406	120.807
5	122.389	135.092	140.892
	122.389	135.092	140.892
6	146.971	145.203	166.755
	146.971	145.203	166.755
7	168.885	168.820	193.711
	168.885	168.820	193.711
8	223.029	263.709	286.325
	223.029	263.709	286.325

Supplementary Table 9. *g*-Tensors for the low-lying Kramers doublets for Dy^{3+} in **3-Dy**.

Kramers doublet		Dy1	Dy2	Dy3
1	g_x	0.00423	0.01492	0.00503
	g_y	0.00837	0.02902	0.00806
	g_z	19.49151	19.24095	19.68294
2	g_x	0.86405	0.16320	1.22502
	g_y	1.09542	0.26337	2.56564
	g_z	17.50331	18.59501	14.52369
3	g_x	0.21328	1.51492	0.13761
	g_y	0.97434	4.63478	1.15670
	g_z	17.59941	13.90572	16.39110
4	g_x	2.22590	8.01559	2.50449
	g_y	3.49025	5.43461	3.41616
	g_z	12.14247	0.96668	11.47954
5	g_x	1.68286	1.14957	0.66293
	g_y	3.40168	2.77558	1.51748
	g_z	14.01526	10.03090	14.19471
6	g_x	0.88140	0.70816	0.52837
	g_y	2.46254	3.34131	2.96868
	g_z	12.83301	14.26587	12.89100
7	g_x	0.51028	2.26081	1.64233
	g_y	2.40652	2.68398	2.57990
	g_z	16.19778	14.97747	16.47459
8	g_x	0.45265	0.13082	0.20210
	g_y	1.49304	0.24894	0.51674
	g_z	17.74837	18.92904	18.72388

Supplementary Table 10. Exchange coupling and dipolar coupling parameters and the spectrum of exchange Kramers doublets in **2-Dy** and **3-Dy** (cm^{-1}).

Interaction		**2-Dy**	**3-Dy**
dipolar*	Dy1-Dy2	–1.116	–1.210
	Dy2- Dy3	–1.202	–1.232
	Dy3- Dy1	–1.110	–1.221
exchange	Dy1- Dy2	–3.014	–1.492
	Dy2- Dy3	–2.878	–1.495
	Dy3- Dy1	–2.991	–1.528
Exchange spectrum	1	0.0000	0.0000
	2	0.0035	0.0261
	3	0.0198	0.0286
	4	3.8459	2.6240

* Only the term of the dipolar interaction is shown here. In the POLY_ANISO calculation, all terms were included.
